# LncRNA H19 promotes adipogenic differentiation disorder by sponging miR-130b-3p to upregulate PPARγ in steroid-induced osteonecrosis of the femoral head

**DOI:** 10.3389/fgene.2025.1529797

**Published:** 2025-04-07

**Authors:** Feifei Lin, Min Yi, Shicheng Zhou, Qingyu Wang

**Affiliations:** Department of Orthopedics of the Second Hospital of Jilin University, Changchun, Jilin, China

**Keywords:** steroid-induced osteonecrosis of the femoral head, H19, miR-130b-3p, PPARγ, bone marrow mesenchymal stem cells

## Abstract

Steroid-induced osteonecrosis of the femoral head (SONFH) is a debilitating condition linked to glucocorticoid-induced adipogenic dysregulation of bone marrow mesenchymal stem cells (BMSCs). While long noncoding RNA H19 has been implicated in differentiation disorders across pathologies, its role in SONFH remains undefined. This study investigated H19’s regulatory mechanism in SONFH progression. We observed significant upregulation of H19 in both femoral head lesions and BMSCs from SONFH patients compared to controls. Knockdown of H19 in SONFH-derived BMSCs suppressed peroxisome proliferator-activated receptor γ (PPARγ) expression, attenuated adipogenic differentiation, and reduced lipid accumulation, as evidenced by decreased Oil Red O staining and FABP4 levels. Mechanistically, H19 acted as a competitive endogenous RNA (ceRNA) by sponging miR-130b-3p, thereby alleviating miR-130b-3p–mediated repression of PPARγ. Luciferase assays confirmed direct binding between miR-130b-3p and H19/PPARγ, while rescue experiments demonstrated that miR-130b-3p inhibition reversed PPARγ downregulation induced by H19 silencing. Our findings reveal a novel H19/miR-130b-3p/PPARγ axis driving adipogenic differentiation of BMSCs in SONFH, positioning H19 as a potential therapeutic target. This study provides critical insights into the epigenetic regulation of BMSC lineage commitment in SONFH pathogenesis, offering new avenues for intervention.

## 1 Introduction

Steroid-induced osteonecrosis of the femoral head (SONFH) is a progressive and destructive orthopedic disorder induced by the long-term, high-dose administration of glucocorticoids (Li et al., 2018; Fu et al., 2019; Xu et al., 2023). Within the collapsed femoral head, there is a notable replacement of bone tissue with adipose tissue, leading to cystic changes. Bone marrow mesenchymal stem cells (BMSCs), with augmented adipogenic potential, not only forfeit their reparative capacity, but also culminate in the catastrophic accumulation of adipocytes and increased intraosseous pressure within the femoral head, further exacerbating the progression of SONFH (Chen et al., 2016; Jiang et al., 2023). Previous studies have revealed that disorders in the adipogenic and osteogenic differentiation of BMSCs play a crucial role in the occurrence and development of SONFH (Wang A. et al., 2018; Chen G. et al., 2020; Kong et al., 2020). However, detailed molecular regulatory mechanisms remain unclear.

Long noncoding RNAs (lncRNAs) play a pivotal role in epigenetic regulation by employing mechanisms such as signal transduction, decoys, guidance, and scaffolding (Mirzadeh Azad et al., 2021; Lin et al., 2022). The lncRNA, H19, plays a multifaceted regulatory role in differentiation disorders through epigenetic, post-transcriptional, and signaling pathway modulation (Wang Q. et al., 2018; Wang et al., 2021; Busscher et al., 2022; Liao et al., 2023). In developmental syndromes, such as Beckwith-Wiedemann syndrome, dysregulation of the H19/insulin-like growth factor 2 (IGF2) imprinting control region leads to H19 downregulation and IGF2 overexpression, driving embryonic cell overgrowth and differentiation defects (Robbins et al., 2012). In cancer, H19 acts as an oncogenic driver by functioning as a competitive endogenous RNA (ceRNA) to sequester tumor-suppressive micro (mi)RNAs, thereby promoting dedifferentiation, metastasis, and chemoresistance in endometrial and colorectal cancers via the miR-612/HOXA10 and Wnt/β-catenin pathways, respectively (Wu et al., 2017; Zhang et al., 2018).

H19 also plays a significant functional role in regulating the osteogenic differentiation of human adipose-derived stem cells. Specifically, this differentiation is facilitated by the suppression of H19 expression, which consequently leads to increased expression of pro-osteogenic genes (Zhou et al., 2021). Moreover, the overexpression of H19 contributes to the downregulation of pro-osteogenic genes (Huang et al., 2017) promotes steatosis, and augments lipid accumulation (Liu et al., 2018). This scenario seems akin to the diminished osteogenic differentiation and augmented adipogenic dysregulation of BMSCs in SONFH; however, the regulatory role of H19 in this context remains to be elucidated.

Here, we conducted a systematic investigation to explore the functional significance of H19 in SONFH. Our study revealed that H19 was significantly overexpressed in both the BMSCs and lesion tissues of patients with SONFH. Suppression of H19 inhibited peroxisome proliferator-activated receptor γ (PPARγ) expression and reduced adipogenic differentiation by directly up-regulating miR-130b-3p. Overall, our data offer an innovative perspective on the regulatory role of H19 in SONFH, suggesting that it is a potential therapeutic target for the treatment of SONFH.

## 2 Methods

### 2.1 Patient specimens

Eight patients with SONFH (1 male and 7 females, 50–74 years of age (mean age of 59.6 ± 7.5 years) and eight patients with femoral neck fracture (FNF) (2 males and 6 females, 57–84 years of age (mean age of 75.0 ± 8.0 years) were enrolled from the Department of Orthopedics, the Second Hospital of Jilin University, China from January 2023 to October 2023. Specimens from patients with SONFH and control subjects with FNF were obtained from individuals undergoing total hip arthroplasty (THA). The diagnosis of SONFH was confirmed preoperatively using radiography and magnetic resonance imaging in accordance with the Association Research Circulation Osseous (ARCO) classification system (Moya-Angeler et al., 2015). Steroid-induced osteonecrosis was defined as a history of taking a mean daily dose of 16.6 mg, or an equivalent maximum daily dose of 80 mg, of prednisolone within 1 year (Koo et al., 2002; Zhang et al., 2014). Patients with concomitant congenital diseases, ethanol consumption, or tumor-related illnesses were excluded from the study. Additionally, none of the patients were taking any medications known to affect bone metabolism. The demographic and clinical characteristics of the patients included in this study are summarized in Table 1.

**TABLE 1:** Characteristics of the patients in this study.

	SONFH (n = 8)	Control (n = 8)	P
Age (years)	62.1 ± 7.2	70.0 ± 7.6	0.067
Gender (M/F)	1/7	2/6	—
CRP (mg/dL)	2.1 ± 1.3	29.6 ± 32.8	**0.044**
ESR (mm/h)	13.9 ± 18.45	30.25 ± 21.5	0.072
BMI (kg/m^2^)	25.5 ± 2.8	23.1 ± 2.1	0.096
ARCO (III/IV)	2/6	—	—
Femoral head collapse (mm)	9.6 ± 2.6	—	—
Harris score	59.2 ± 4.6	—	—

CRP, C-reaction protein; ESR, erythrocyte sedimentation rate; BMI, Body Mass Index; ARCO, association research circulation osseous.

The bold values indicate P < 0.05.

### 2.2 BMSC isolation and culture

BMSCs were isolated from the bone marrow of proximal femurs of patients by density gradient centrifugation (Colter et al., 2000). Cells were cultured in Dulbecco’s modified Eagle’s medium (Gibco, Gaithersburg, MD, United States) supplemented with 10% fetal bovine serum (FBS, Gibco) and incubated at 37°C with 5% CO2. The culture medium was replenished every 3 days. Upon reaching 90% confluence, the BMSCs were trypsinized and subcultured in new plates. The cells were expanded and used for experiments at passage 3.

### 2.3 RNA extraction

Total RNA was extracted using TRIzol Reagent (Invitrogen, Carlsbad, CA, United States) in accordance with the manufacturer’s instructions. The RNA concentration was quantified using an Agilent ND-1000 (Santa Clara, CA, United States).

### 2.4 Cell transfection

The human H19 shRNA vector (sh-H19), overexpression vector (ov-H19), miR-130b-3p mimic and inhibitor were purchased from GenePharma (Shanghai, China). Lipofectamine 3000 (Thermo Fisher Scientific, Waltham, MA, United States) was used for cell transfection according to the manufacturer’s instructions. Cells were harvested 48 h post-transfection for the quantitative real-time polymerase chain reaction (qRT-PCR) analysis, with each experiment being conducted in triplicate.

### 2.5 qRT-PCR

Total RNA was reverse-transcribed into cDNA using the PrimeScript RT Reagent Kit with gDNA Eraser (TaKaRa Bio, Beijing, China) following the manufacturer’s guidelines. qRT-PCR was performed using FastStart Universal SYBR Green Master Mix (Roche, Basel, Switzerland) on an Applied Biosystems 7500 Fast Real-Time PCR System (Foster City, CA, United States) according to the manufacturer’s instructions. Data were normalized against the expression of glyceraldehyde-3-phosphate dehydrogenase, and the relative expression levels of each gene were calculated using the 2^−ΔΔCT^ method. All experiments were conducted in triplicate. Primer sequences used in this study are listed in Table 2.

**TABLE 2:** Primers used for real-time RT-PCR analysis.

Gene	Primer sequences
H19-F	5′- TCT​GGC​AGG​AGT​GAT​GAC​GG -3′
H19-R	5′- CAG​GAG​AGT​TAG​CAA​AGG​TG -3′
PPARγ-F	5′- GAG​CCC​AAG​TTT​GAG​TTT​GC-3′
PPARγ-R	5′- CTG​TGA​GGA​CTC​AGG​GTG​GT-3′
FABP4-F	5′- CAG​GAA​AGT​GGC​TGG​CAT​GGC-3′
FABP4-R	5′-GCT​CTC​TCA​TAA​ACT​CTC​GTG​GAA​GTG-3′
GAPDH-F	5′- CGG​ACC​AAT​ACG​ACC​AAA​TCC​G-3′
GAPDH-R	5′- AGC​CAC​ATC​GCT​CAG​ACA​CC-3′
miR-130b-3p-F	5′- GGG​CAG​TGC​AAT​GAT​GAA​A -3′
miR-130b-3p-R	5′- ACA​GAC​CCA​GCC​AAC​AAA​TA -3′
U6-F	5′- GCT​TCG​GCA​GCA​CAT​ATA​CTA​AAA​T -3′
U6-R	5′- CGC​TTC​ACG​AAT​TTG​CGT​GTC​AT -3′

GAPDH, glyceraldehyde-3-phosphate dehydrogenase.

### 2.6 Western blotting

Total proteins of BMSCs were extracted using the Total Protein Extraction Kit (Signalway Antibody LLC, Maryland, United States) according to the manufacturer’s instructions. Equal amounts of protein were resolved by sodium dodecyl sulfate–polyacrylamide gel electrophoresis and transferred to nitrocellulose membranes via electroblotting. After blocking with 5% skimmed milk, the membrane was incubated with primary antibodies at 4°C overnight, followed by incubation with anti-rabbit secondary antibody (Boster Biological Technology, Wuhan, China). Ultimately, the protein levels were visualized via using a high sensitive ECL luminescence reagent (Sangon Biotech Co. Ltd., Shanghai, China). The primary antibody PPARγ, FABP4 and GAPDH) were purchased from Boster Biological Technology (Boster Biological Technology, Wuhan, China). All experiment was repeated three times.

### 2.7 Osteogenic and adipogenic differentiation

Osteogenic differentiation was induced by culturing BMSCs in an osteogenic medium supplemented with 10% FBS, 0.1 μmol/L dexamethasone, 10 μmol/L β-glycerophosphate, 10 μmol/L glutamine, and 50 μg/mL ascorbate (Cyagen Biosciences, Guangzhou, China). For adipogenic differentiation, BMSCs were cultured in an adipogenic medium containing 10% FBS, 1 μmol/L dexamethasone, 100 μg/mL 3-isobutyl-1-methylxanthine, 2 μg/L insulin, 1 μmol/L rosiglitazone, and 10 μmol/L glutamine (Cyagen).

### 2.8 Oil Red O staining and quantification

Oil Red O (Beyotime, Beijing, China) staining was used to evaluate the accumulation of intracellular lipids following adipogenic differentiation. Cells were fixed in 4% neutral-buffered formalin for 30 min, washed with 3% isopropanol, incubated with freshly filtered Oil Red O staining solution for 1 h, and rinsed with double-distilled water. For quantitative analysis, isopropyl alcohol was added to the stained culture dishes and the optical density values were measured at 490 nm. ImageJ software (National Institutes of Health, Bethesda, MD, United States) was used to enumerate the cells exhibiting Oil Red O staining.

### 2.9 Bioinformatics analysis

LncRNA-miRNA interactions were predicted using Diana tools (Karagkouni et al., 2020) and StarBase (Li et al., 2014). miRNA interaction with mRNAs was predicted using miRWalk (Sticht et al., 2018) and StarBase. A regulatory pattern diagram was constructed using FigDraw.

### 2.10 Luciferase reporter assay

Wild-type (WT) or mutant (MT) MNX1-H19 fragments containing the miR-130b-3p binding site were introduced into a pGL3-basic vector (Promega, Madison, WI, United States). For the luciferase assay, 293T cells were transfected simultaneously with the miR-130b-3p mimic or the corresponding nonsense control. Luciferase activity was detected 48 h post-transfection using the Luciferase Reporter Gene Detection Kit (Promega), according to the manufacturer’s instructions. All experiment was repeated three times.

### 2.11 Statistical analysis

Data are presented as means ± standard deviation. All statistical analyses were performed using SPSS version 20.0 software (IBM, Chicago, IL, United States). Comparisons between groups were performed using the unpaired Student’s t-test. Statistical significance was set at P < 0.05.

## 3 Results

### 3.1 H19 is upregulated in femoral head lesions and BMSCs from SONFH patients

**FIGURE 1:**
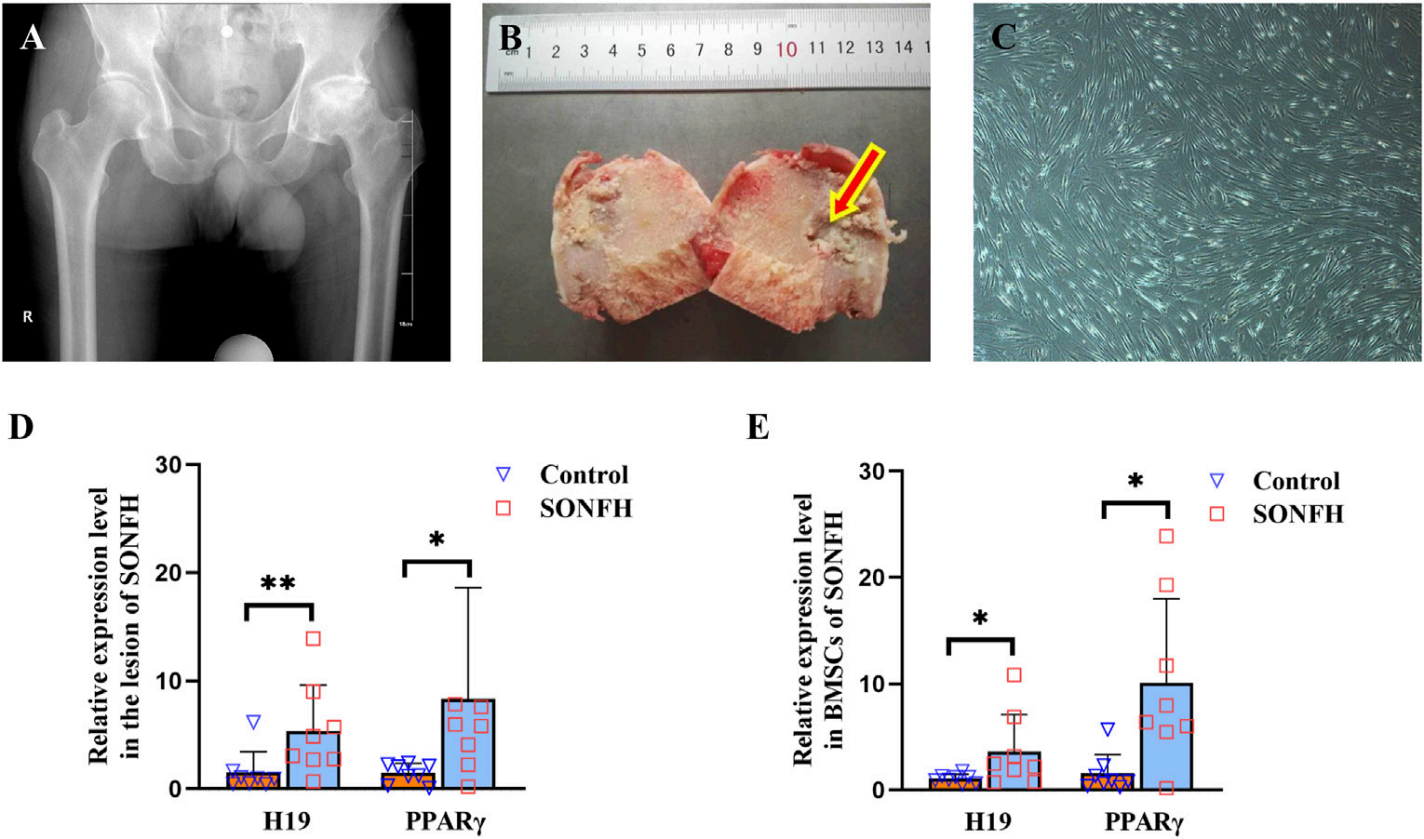
H19 and PPARγ are upregulated in the femoral head and BMSCs of patients with SONFH. **(A, B)** X-ray photo and pathological structure of the femoral head from an ARCO stage V SONFH patient. The images show alterations in the morphology of the femoral head, characterized by collapse and flattening, as well as radiographic signs indicative of hip osteoarthritis. **(C)** Morphology of BMSCs from a patient with SONFH. **(D, E)** Expression levels of H19 and PPARγ in the femoral head and BMSCs from a patient with SONFH. All experimental procedures were performed in triplicate with internal normalization to GAPDH expression levels. The relative expression levels of each gene were analyzed using the 2^−△△Ct^ method (n = 8, all data are shown as the mean ± SD of three independent experiments, *p < 0.05, **p < 0.01).

X-ray imaging of patients with SONFH at ARCO stage V revealed alterations in the morphology of the femoral head, characterized by collapse and flattening, as well as radiographic signs indicative of hip osteoarthritis (Figure 1A). During THA, we meticulously harvested the femoral head from patients with SONFH and carefully extracted tissue from the osteonecrotic zone (Figure 1B). The femoral head tissue of patients with FNF served as the control group. During surgery for SONFH and FNF, the bone marrow was collected and BMSCs were subsequently extracted and cultured. The morphology of BMSCs derived from patients with SONFH is shown in Figure 1C. The expression levels of H19 and PPARγ were found to be abnormally and significantly upregulated in the femoral head tissues (Figure 1D) and BMSCs (Figure 1E) of SONFH patients.

### 3.2 H19 participates in increased adipogenesis of BMSCs and positively regulates PPARγ expression in SONFH

**FIGURE 2:**
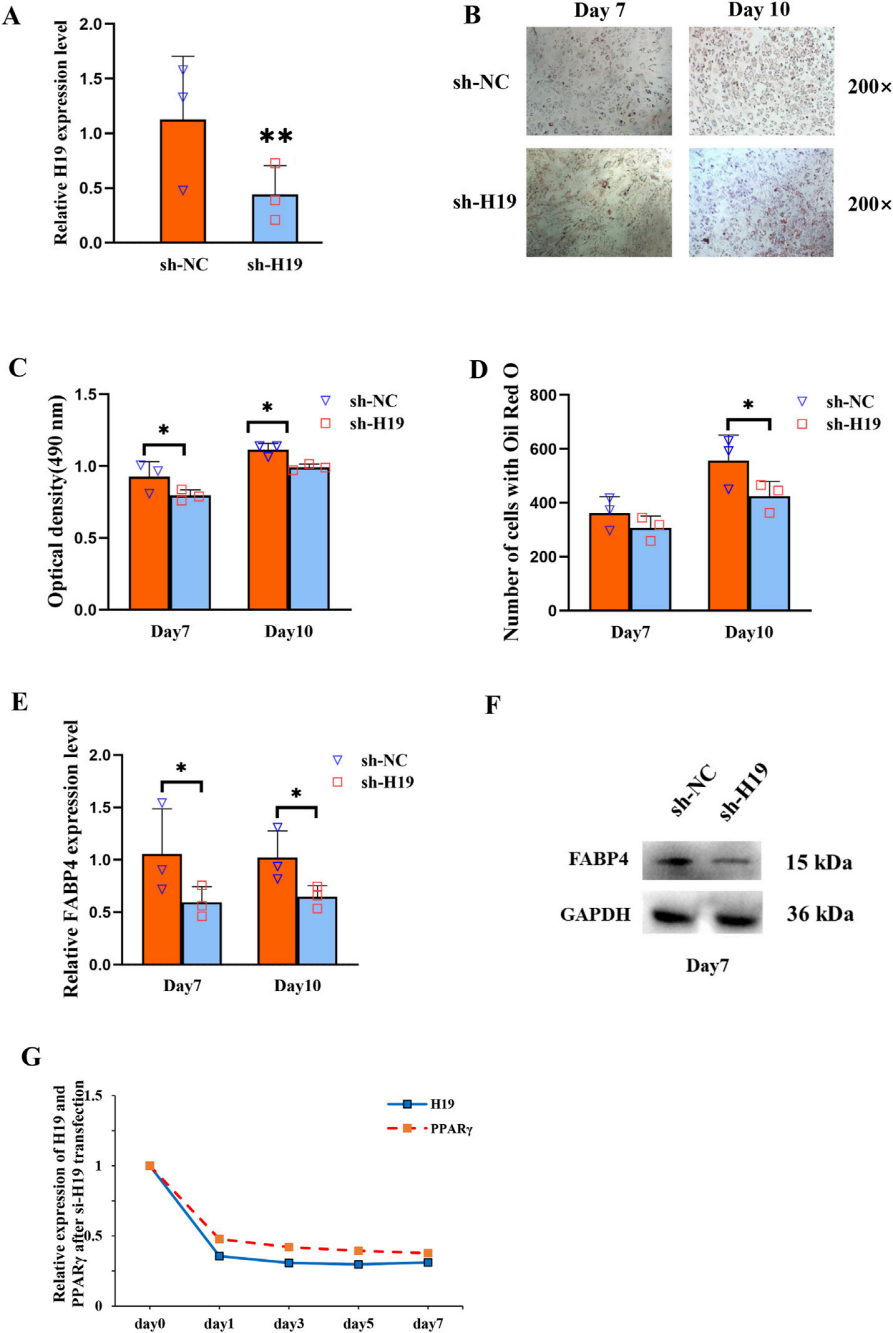
H19 participates in increased adipogenesis of BMSCs and positively regulates PPARγ expression in SONFH. **(A)** Expression level of H19 in BMSCs after transfection with sh-H19. **(B)** Oil Red O staining (200×) of BMSCs after transfection with sh-H19. **(C, D)** Quantification of Oil Red O staining (200×) of BMSCs after transfection with sh-H19. **(E, F)** The expression of fatty acid-binding protein 4 (FABP4) was detected by qRT-PCR and Western blot after knocking-down of H19. **(G)** The expression levels of H19 and PPARγ 1 week following the knockdown of H19. (n = 3, all data are shown as the mean ± SD of three independent experiments, *p < 0.05, **p < 0.01).

To investigate the regulatory role of H19, we knocked it down using H19-specific shRNA in BMSCs isolated from patients with SONFH. Following transfection with the H19 shRNA vector, there was significant downregulation of H19 expression (Figure 2A). Furthermore, Oil Red O staining showed that the adipogenic capacity of these transfected BMSCs was reduced (Figure 2B). This trend was corroborated by the quantitative analysis of Oil Red O staining, which revealed that, on days 7 and 10 post adipogenic induction, the staining intensity in the SONFH group was notably lower than that in the control group (Figures 2C, D). Following H19 knockdown, the lipid metabolism marker, fatty acid-binding protein 4 (FABP4), exhibited a marked decrease (Figures 2E, F). The expression levels of both H19 and PPARγ were reduced concurrently within a week after the knockdown of H19 (Figure 2G).

### 3.3 H19 acts as a miR-130b-3p sponge, and PPARγ can be directly targeted by miR-130b-3p

**FIGURE 3:**
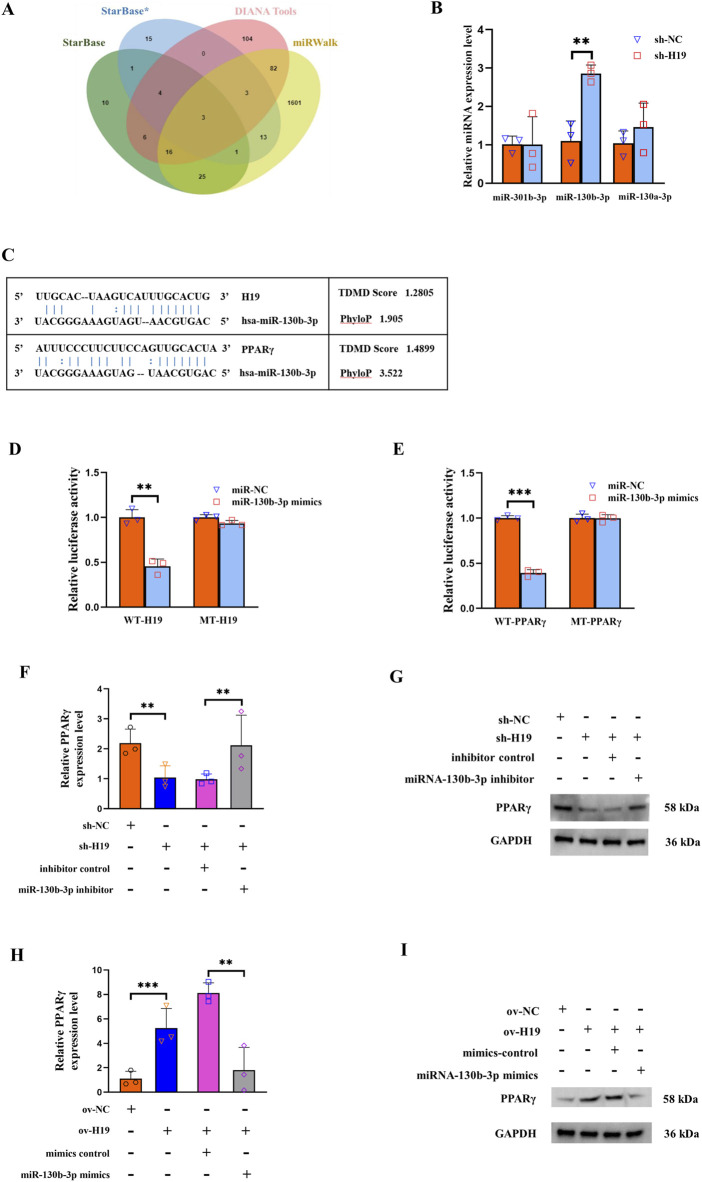
H19 modulates PPARγ expression through miR-130b-3p. **(A)** Three databases, StarBase, DIANA tools, and miRWalk, were used to predict candidate micro (mi)RNAs as shown in the Venn diagram. **(B)** Expression levels of three candidate miRNAs (miR-301b-3p, miR-130b-3p, and miR-130a-3p) after knockdown of H19. **(C)** The binding sites of miR-130b-3p with H19 and PPARγ. **(D)** Effect of miR-130b-3p on the luciferase activity of wild-type (WT)-H19 and mutant (MT)-H19 reporter systems. **(E)** Effect of miR-130b-3p on the luciferase activity of WT-FABP4 and MT-FABP4 reporter systems was detected via luciferase reporter assay. **(F, G)** The expression of PPARγ is significantly decreased upon knocking down H19. This reduction could be reversed through co-transfection with a miR-130b-3p inhibitor. **(H, I)** The expression of PPARγ was significantly augmented when H19 was upregulated. This elevation in PPARγ expression could be counteracted by co-transfecting with a miR-130b-3p mimic. (n = 3, all data are shown as the mean ± SD of three independent experiments, **p < 0.01, ***p < 0.001).

It is well-established that lncRNAs can serve as miRNA “sponges,” thereby inhibiting interactions with their miRNA targets during post-transcriptional regulation. Utilizing the StarBase and DIANA tools, we predicted the miRNAs that could bind to H19, while those that could binding to PPARγ were forecasted using StarBase and miRWalk. The final intersection set included miR-301b-3p, miR-130b-3p, and miR-130a-3p (Figure 3A). To confirm whether H19 regulated the expression of these candidate miRNAs, we performed a qRT-PCR analysis of H19-silenced BMSCs. The results revealed that only miR-130b-3p was significantly upregulated following the reduced expression of H19 (Figure 3B). The binding sites of miR-130b-3p with H19 and PPARγ are shown in Figure 3C.

To verify the binding site of H19/PPARγ and miR-130b-3p, we conducted a luciferase reporter assay. This assay demonstrated that 293T cells overexpressing miR-130b-3p had lower WT-H19 but not MT-H19 luciferase activity (Figure 3D). Similarly, the WT-PPARγ reporter activity decreased upon miR-130b-3p overexpression, with no significant change observed in MT-PPARγ group (Figure 3E). For the rescue experiments, the expression of PPARγ was significantly diminished when H19 was knocked down. This decrease in PPARγ expression could be effectively reversed by co-transfection with an inhibitor of miR-130b-3p (Figures 3F, G). In contrast, the expression of PPARγ was significantly increased when H19 was overexpressed. This rise in PPARγ expression could be counteracted by co-transfecting with the miR-130b-3p mimic (Figures 3H, I).

**FIGURE 4:**
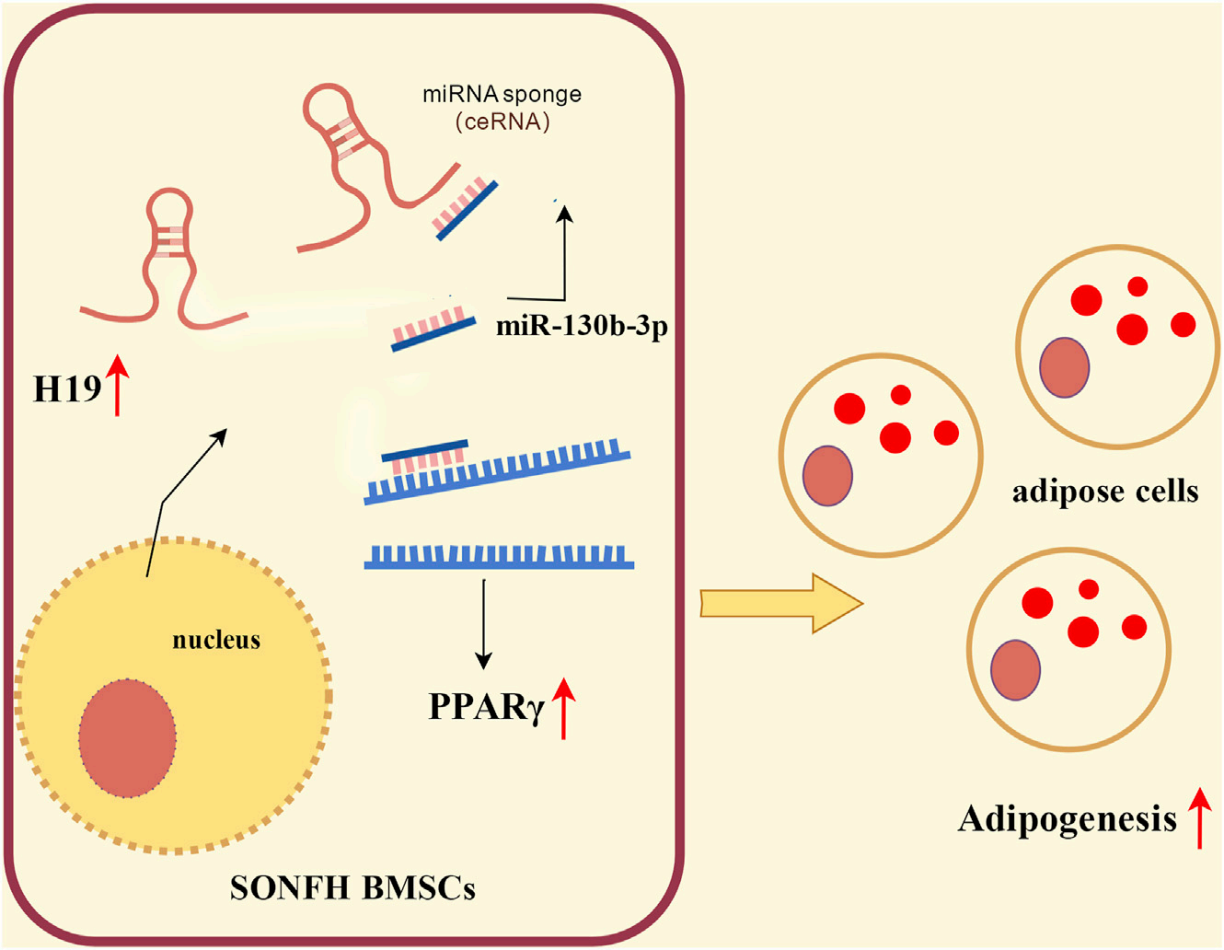
Schematic illustrating the regulatory effect of H19 in SONFH-BMSCs.

Collectively, these findings demonstrate that upregulating H19 expression in SONFH-BMSCs promotes PPARγ expression via competitive binding to miR-130b-3p, which subsequently enhances adipogenic differentiation of BMSCs. This aberrant adipogenic commitment exacerbates SONFH pathogenesis. This mechanistic cascade is schematically summarized in Figure 4.

## 4 Discussion

The misuse of corticosteroid hormones is one of the primary factors contributing to femoral head necrosis (Wang X. et al., 2018; Huang et al., 2021). Steroid-induced endothelial dysfunction disrupts the blood supply to the femoral head, which results in the progressive and robust upregulation of osteoclast-related proteins, and localized bone tissue ischemia and necrosis (Maruyama et al., 2018; Chen K. et al., 2020).

The destruction of bone cells, coupled with an imbalance between osteogenic and osteoclastic activities, ultimately leads to the degradation and collapse of the bone structure (Piuzzi et al., 2019). During this process, the disruption of the differentiation equilibrium in BMSCs represents a substantial pathological change (Powell et al., 2011; Chang et al., 2020). BMSCs with enhanced adipogenic potential not only forfeit their reparative capacity, but also culminate in the catastrophic accumulation of adipocytes and increased intraosseous pressure within the femoral head, further exacerbating the progression of SONFH. In-depth investigations into the mechanisms underlying the differentiation disorders of SONFH-BMSCs are pivotal for a more comprehensive understanding of the pathogenesis of SONFH.

As noted earlier, lncRNAs play an essential role in epigenetic regulation through numerous mechanisms (Wang and Chang, 2011; Mirzadeh Azad et al., 2021). In recent years, extensive efforts have been directed toward elucidating the differential expression profiles of various non-coding RNAs, including lncRNAs and miRNAs, in SONFH-BMSCs (Table 3). Most studies have shown that lncRNAs influence the differentiation lineage of BMSCs by modulating post-transcriptional mRNA levels via a ceRNA mechanism. Our previous work determined the lncRNA expression profile in human SONFH-BMSCs, leading to the hypothesis that MALAT1 modulates the expression of DKK1, thereby influencing the osteogenic and adipogenic differentiation of BMSCs (Wang X. et al., 2018). Wu et al. reported that the lncRNA, FGD5-AS1, regulates BMSC proliferation and apoptosis by affecting the miR-296-5p/STAT3 axis in SONFH (Wu et al., 2022). Han et al. demonstrated that H19-hsa-miR-519b-3p/hsa-miR-296-5p-ankylosis protein homolog (ANKH) and the lncRNA, c9orf163-hsa-miR-424-5p-CCNT1, may play important roles in osteonecrosis during femoral head development (Han and Li, 2021). Our study shows that H19 promotes the adipogenic differentiation of BMSCs and aggravates the progression of SONFH through the miR-130b-3p/PPARγ axis. Because PPARγ is recognized as a transcription factor that facilitates adipogenic differentiation while inhibiting osteogenic differentiation (Cao, 2011; Berhouma et al., 2013), our findings substantiated that the dysregulation of H19 contributes to disrupting the equilibrium between adipogenic and osteogenic differentiation in SONFH, as well as illuminating its regulatory role. These newly identified SONFH-associated lncRNAs offer novel insights, not only for further elucidating the molecular regulatory mechanisms of SONFH, but also for providing novel molecular markers and therapeutic targets for the diagnosis and treatment of femoral head necrosis.

**TABLE 3:** Functional characterization of the ncRNAs in SONFH.

Non-coding RNAs	Name	Expression	Functional role	Target miRNAs	Target genes	Sample	References
circRNA	circHGF	Up	Suppress proliferation and osteogenic differentiation of BMSCs	miR-25-3p	SMAD7	hBMSCs (7 Male and 3 Female)	Feng et al. (2022)
circRNA	circ_0058122	Up	increase dex-mediated HUVEC apoptosis	miR-7974	IGFBP5	Femoral head tissues (3 Male and 7 Female); HUVECs cells	Yao et al. (2022)
LncRNA	LINC00473	Down	promote osteogenesis and suppress the adipogenesis of BMSCs	miR-23a-3p	LRP5	hBMSCs	Xu et al. (2022)
LncRNA	FGD5-AS1	Up	promote cell proliferation and restrain apoptosis	miR-296-5p/	STAT3	hBMSCs	Wu et al. (2022)
LncRNA	NORAD	Down	promotion of proliferation and differentiation, and inhibition of apoptosis	miR-26a-5p	—	hBMSCs (20 patients)	Fu et al. (2021)
LncRNA	RP11-154D6	Down	promote BMSCs osteogenic differentiation and inhibit adipogenic differentiation	miR-30a	—	hBMSCs (7 patients)	Xiang et al. (2019)

H19 was the first lncRNA to be identified. It possesses a multitude of diverse biological functions and participates in the regulation of cellular proliferation, differentiation, and metabolism (Zhu et al., 2024). Previous studies demonstrated that H19 is involved in fat accumulation and regulation. The expression of H19 is augmented by fatty acids in hepatocytes and high-fat diet-induced fatty liver, with the overexpression of H19 promoting steatosis and enhancing lipid accumulation (Liu et al., 2018). Although Han et al. proposed a ceRNA network whereby H19 could act as a ceRNA for hsa-miR-519b-3p and hsa-miR-296-5p in ANKH (Han and Li, 2021), this has yet to be substantiated by relevant experimental validation. In our study, H19 was aberrantly upregulated in both osteonecrotic femoral head tissues and BMSCs. The relationship between H19 and miR-130b has been reported to regulate keratinocyte differentiation (Li et al., 2017) and potentiate the effect of praziquantel on liver function (Ma et al., 2023). However, this relationship has not yet been explored in the context of SONFH. Based on these findings, we hypothesized that H19 exerts a regulatory effect on SONFH progression by functioning as a sponge for miR-130b-3p. Our current investigation revealed that miR-130b-3p expression was increased following H19 knockdown. Additionally, we observed an inverse correlation between PPARγ and miR-130b-3p expression. Through bioinformatics analyses and rescue experiments, we substantiated that miR-130b-3p could interact with both H19 and PPARγ. Collectively, our results suggest that H19 may modulate PPARγ expression by targeting miR-130b-3p.

Previous research has demonstrated that corticosteroids have the capacity to upregulate the expression of PPARγ in both rodent and human BMSCs, thereby fostering adipogenic differentiation (Sheng et al., 2007). Our previous research also identified an association between gene variants of the transcription factor PPARγ and the development of osteonecrosis of the femoral head in the Chinese population (Song et al., 2017). Several studies have also reported the abnormal expression and related regulatory effects of PPARγ in osteonecrosis of the femoral head (Zhao et al., 2019; Cui et al., 2022) (Fu et al., 2016). The results of the current study revealed that PPARγ expression was notably elevated in both the femoral head tissues and BMSCs of patients with SONFH. Additionally, we found that miR-130b-3p regulated adipogenic differentiation by targeting PPARγ in the context of SONFH.

In summary, our findings reveal that elevated H19 expression is a characteristic molecular alteration in SONFH, and that H19 fosters BMSC adipogenic differentiation by enhancing PPARγ activity through the suppression of miR-130b-3p. Collectively, our study substantiates the regulatory function of lncRNAs in SONFH progression, positioning H19 as a pivotal and novel molecular target.

Our study has some limitations. First, larger cohort studies are required to validate these results and account for potential confounding variables such as age, sex, and glucocorticoid dosage heterogeneity. Second, H19’s therapeutic potential in animal models of steroid-induced osteonecrosis needs to be evaluated. Additionally, a more comprehensive analysis of H19-associated networks could further elucidate its multifaceted roles in BMSC differentiation and bone homeostasis.

## 5 Conclusion

This study confirmed that the aberrant upregulation of H19 contributes to abnormal adipogenic differentiation in SONFH by functioning as a molecular sponge for miR-130b-3p and subsequently upregulating PPARγ. These findings offer an innovative perspective on the treatment of SONFH.

## Data Availability

The raw data supporting the conclusions of this article will be made available by the authors, without undue reservation
